# Differentiating Branch Duct and Mixed IPMN in Endoscopically Collected Pancreatic Cyst Fluid via Cytokine Analysis

**DOI:** 10.1155/2012/247309

**Published:** 2012-12-25

**Authors:** Linda S. Lee, Andrew M. Bellizzi, Peter A. Banks, Nisha I. Sainani, Vivek Kadiyala, Shadeah Suleiman, Darwin L. Conwell, Joao A. Paulo

**Affiliations:** ^1^Center for Pancreatic Disease and Division of Gastroenterology, Hepatology and Endoscopy, Brigham and Women's Hospital and Department of Medicine, Harvard Medical School, 75 Francis Street, Boston, MA 02115, USA; ^2^Department of Pathology, University of Iowa Hospitals and Clinics, Iowa City, IA 52242, USA; ^3^Department of Radiology, Brigham and Women's Hospital, Boston, MA 02115, USA; ^4^Department of Pathology, Boston's Children Hospital, Boston, MA 02115, USA; ^5^Proteomics Center, Boston's Children Hospital, Boston, MA 02115, USA

## Abstract

*Background*. Differentiating branch duct from mixed intraductal papillary mucinous neoplasm (BD-IPMN) is problematic, but clinically important as mixed IPMNs are managed surgically, while some BD-IPMN may be followed. Inflammatory mediator proteins (IMPs) have been implicated in acute and chronic inflammatory and malignant pancreatic diseases. *Aim*. To compare IMP profile of pancreatic cyst fluid collected endoscopically from BD-IPMN and mixed IPMN. *Methods*. Pancreatic cyst fluid from ten patients (5 BD-IPMN and 5 mixed IPMN) was collected by endoscopic ultrasound-guided fine needle aspiration or endoscopic retrograde cholangiopancreatography. Concentrations of 89 IMPs in these samples were determined using a multiplexed bead-based microarray protein assay and compared between BD-IPMN and mixed IPMN. *Results*. Eighty-six of 89 IMPs were detected in at least one of the 10 samples. Fourteen IMPs were detected only in mixed IPMN, while none were only in BD-IPMN. Of these, TGF-**β**1 was most prevalent, present in 3 of 5 mixed IPMNs. Seventy-two IMPs were detected in both BD-IPMN and mixed IPMNs. Of these, only G-CSF (*P* < 0.05) was present in higher concentrations in mixed IPMNs. *Conclusion*. TGF-**β**1 and G-CSF detected in endoscopically collected pancreatic cyst fluid are potential diagnostic biomarkers capable of distinguishing mixed IPMN from BD-IPMN.

## 1. Introduction

Many pancreatic cystic lesions have malignant potential, including branch duct and mixed intraductal papillary mucinous neoplasms (BD-IPMNs). As the malignant risk is substantially greater for mixed IPMN than BD-IPMN, current management of mixed IPMN is surgical, while many BD-IPMN may be managed conservatively. Therefore, accurately distinguishing them has important clinical implications. Diagnosis of these lesions relies mainly on the combination of diagnostic imaging and analysis of cyst fluid obtained during endoscopic ultrasound-guided fine needle aspiration (EUS-FNA). While EUS-FNA is safe [1], the diagnostic accuracy of cytology, carcinoembryonic antigen (CEA), amylase, and DNA markers from cyst fluid is limited [2, 3]. Traditional biochemical cyst fluid analysis generally requires 0.5–1 mL of cyst fluid. Particularly for small pancreatic cysts, EUS-FNA generally yields less than the essential minimum quantity, which limits the ability to classify these lesions. Therefore, better diagnostic biomarkers for pancreatic cystic lesions are needed. 

Differentially expressed inflammatory mediator proteins (IMPs) may serve as diagnostic biomarkers for pancreatic cystic neoplasms. IMPs, which include cytokines, chemokines, and growth factors, are commonly associated with acute and chronic disease states. Cytokines are low molecular weight regulatory proteins produced by various cell types particularly during cellular stress events. Generally released in picomolar amounts, their concentration can increase over 1000-fold during physiological stress, such as trauma or infection [4]. Chemokines are a superfamily of small chemoattractant cytokines (8–10 kDa) which guide the migration of cells via corresponding chemokine receptors [5]. These proteins attract neutrophils, monocytes, and other circulating effector cells to sites of infection or tissue damage [6]. Similar to cytokines, many chemokines are considered proinflammatory. Other chemokines are considered homeostatic, involved in controlling the migration of cells during normal tissue maintenance or development [5]. 

The simultaneous analysis of numerous IMPs can be performed in a single experiment with a suspension microarray using IMP-specific capture antibodies coupled to color-coded microspheres. Current IMP microarrays are both sensitive to low concentrations of cytokines and amenable to high-throughput analysis [7], making this technique ideal for biomarker screening. Although the primary clinical use of this technology is in the analysis of urine and blood, microarray-based approaches may also be applied to proximal body fluids, such as pancreatic cyst fluid. We previously performed an analogous IMP microarray-based analysis of pancreatic fluid collected during secretin-stimulated endoscopic pancreatic function testing to characterize IMPs in chronic pancreatitis [8].

The primary objective of our exploratory investigation is to compare IMP profiles in endoscopically collected pancreatic cyst fluid aspirates of known BD-IPMN and mixed IPMN using a multiplexed IMP-targeted microarray. 

## 2. Materials and Methods 

### 2.1. Study Population

The study was designed to analyze IMPs in endoscopically collected pancreatic cyst fluid using a multiplexed suspension microarray assay in an academic center. This protocol was approved by the Partners Institutional Review Board. The study population included adult patients referred to the Center for Pancreatic Diseases at Brigham and Women's Hospital for evaluation of pancreatic cystic lesions. All subjects underwent the following: (1) comprehensive history and physical examination, (2) review of radiologic data, and (3) EUS-FNA and/or endoscopic retrograde cholangiopancreatography (ERCP).

**Figure 1:**
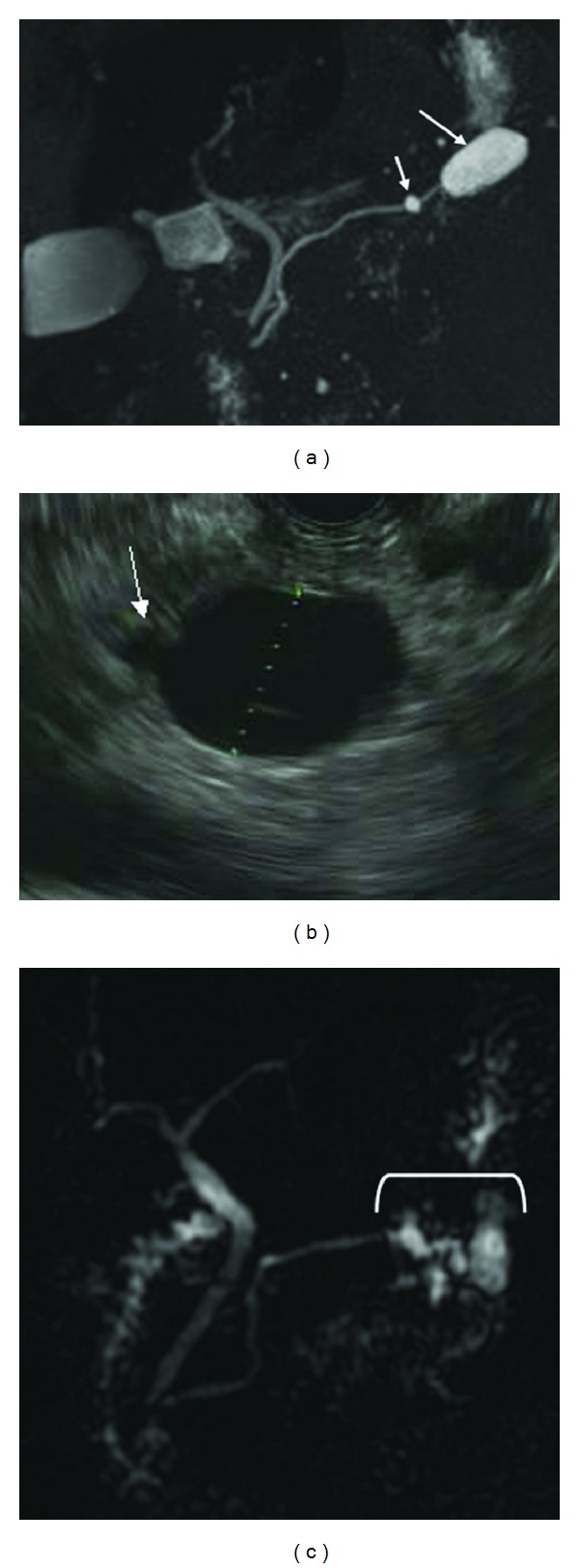
Imaging of BD-IPMN and mixed IPMN. (a) MRI of BD-IPMN: arrow points to communication between BD-IPMN and normal main pancreatic duct. (b) EUS of BD-IPMN: arrow points to communication between cyst and main pancreatic duct. (c) MRI of mixed IPMN: arrow points to diffusely massively dilated main pancreatic duct.

Only patients with diagnoses of BD-IPMN and mixed IPMN were included. Definitive diagnosis was obtained from a combination of methodologies: a physician review of the patient medical history with radiologic imaging, endoscopic findings, and/or surgical pathology. A single abdominal radiologist (NS), blinded to the official radiology report, reviewed the radiologic studies, which included both abdominal computed tomography (CT) and magnetic resonance cholangiopancreatography (MRCP). By radiology and/or EUS, BD-IPMN was defined as a unilocular or multiloculated pancreatic cyst with smooth or loculated margins with a demonstrable communication (short neck or long channel) to a nondilated main pancreatic duct [9, 10] (Figures 1(a) and 1(b)). Absence of a discernable communication does not exclude BD IPMN since the communication can be diminutive or blocked by mucus and hence not visualized. Mixed IPMN was defined as a cystic lesion with ductal communication and main pancreatic duct dilation greater than or equal to 5 mm (Figure 1(c)). Histologically, BD-IPMN and mixed IPMN were defined as a grossly visible, noninvasive, mucin-producing papillary epithelial neoplasm arising from the branch ducts or both branch and main pancreatic ducts, respectively [11]. ERCP findings diagnostic of at least main duct involvement in IPMN include the presence of a “fish mouth papilla,” indicating the presence of mucin within the main pancreatic duct and visualization of a fish egg appearance in the main pancreatic duct during pancreatoscopy [12].

### 2.2. Experimental Workflow

The overall analysis proceeded as follows: (A) EUS-FNA or ERCP sample collection, (B) particulate removal via centrifugation, (C) multiplexed IMP microarray assays, and (D) statistical analysis of the resulting profiles.

### 2.3. Endoscopic Ultrasound-Guided Fine Needle Aspiration (EUS-FNA) and ERCP

Endosonography was performed with a curvilinear echoendoscope (Olympus GF-UC(T)140P-OL5; Olympus America Inc., Center Valley, PA) using Aloka SSD-Alpha 5 and Alpha 10 (Olympus America Inc., Center Valley, PA) processors. Curvilinear echoendoscopes are modified, oblique forward-viewing instruments with curved linear ultrasound transducers providing real-time visualization of the aspiration needle. In brief, after obtaining informed consent and administration of intravenous conscious sedation, the echoendoscope was advanced into the upper gastrointestinal tract, the target lesion located, and FNA of the cystic lesion performed using a 22-gauge adjustable needle (EZ Shot, Olympus, Center Valley, PA). Aspirates were divided into three aliquots for (1) biochemical analysis of CEA and amylase, (2) IMP assay, and (3) cytologic evaluation with fluid placed into Cytolyt preservative (Cytyc, Boxborough, MA). Samples were stored at −80°C until IMP analysis (see Section 2.4). Antibiotic prophylaxis was administered during the procedure and for 3 days following the procedure.

The ERCP procedure proceeded in a similar manner to EUS with the exception of using a duodenoscope (Olympus TJF-160VF; Olympus America Inc., Center Valley, PA) and cannula (Tandem XL M00535700; Boston Scientific, Natick, MA) to cannulate the pancreatic duct. Pancreatic duct fluid was aspirated through the cannula and samples sent for analyses.

### 2.4. Pancreatic Cyst Fluid IMP Microarray Analysis

A suspension microarray assay was used to measure the concentration of 89 IMPs in pancreatic cyst fluid samples from 10 individuals. We selected this 89-cytokine panel as it was the most comprehensive panel available at the time of this study. A list of the IMPs investigated with their corresponding abbreviations is provided in Supplementary Table 1 available online at doi:10.1155/2012/247309. Unlike mass spectrometry-based proteomic assays of pancreatic fluids [13–16], suspension microarray assays require only minimal sample preparation of centrifugation to remove particulates. Immediately following fluid collection, samples were aliquoted into 1.5 mL microtubes and centrifuged on an Eppendorf centrifuge 5415R at 4°C and 10,000 ×g to remove particulates. The supernatant was transferred into a new tube and stored at −80°C prior to analysis.

Immediately prior to the microarray analysis, the concentration of known standards was determined by a 5-parameter logistic regression algorithm with analysis of the median fluorescence intensity readings on an 8-point protein standard curve. This procedure ensured that the reading was within the linear range of the assay. The fluorescence intensity values of the standards were treated as unknowns, and the concentration of each standard was calculated using the derived regression equation. The ratio of the calculated value to the expected value of this standard was determined. A ratio between 70 and 130% for each standard indicated a good fit. If fluorescence intensity values of samples plateaued or were outside the range of standard curves, a retest with diluted samples was performed to ensure that the fluorescence intensity measurement of unknown samples fell inside the linear range of standard curves. 

Levels of IMPs in cyst fluid were determined using microsphere-based suspension microarray technology (AssayGate, Ijamsville, MD) [17]. The microarray analysis was performed according to previously published methods [18–20]. In brief, multiple analytes in a single aliquot (75 *μ*L) of pancreatic fluid were simultaneously quantified with Bio-Plex 200 Bead Reader System (Bio-Rad, Hercules, CA). Microparticles were conjugated previously to differing concentrations of two fluorophores to generate distinct bead sets. Each bead set was coated with a capture antibody specific for one analyte. Captured analyte was detected using a biotinylated detection antibody and streptavidin-phycoerythrin conjugate. The bead analyzer was a dual laser, flow-based, sorting, and detection platform. One laser was bead specific and determined which analyte was being detected. The second laser determined the magnitude of phycoerythrin-derived signal, which is directly proportional to the amount of analyte bound. No more than 75 *μ*L of pancreatic cyst fluid was used for each assay, and each sample was tested in duplicate.

### 2.5. Statistical Analysis

IMP concentrations were expressed in picograms per milliliter (pg/mL) of pancreatic cyst fluid and analyzed by the Kruskal-Wallis one-way analysis of variance by a rank test for two samples using SAS 9.2 (Cary, NC). A *P* value < 0.05 was considered statistically significant. For the purpose of this exploratory analysis, a *P* value < 0.1 was considered a trend warranting further investigation. The Bonferroni or Benjamini-Hochberg correction method is generally used to account for multiple testing of collected samples, but was not used in our study as it is not required for exploratory data analysis [21]. 

## 3. Results

### 3.1. Patient Characteristics

The demographics and clinical characteristics of the 10 study subjects are shown in Table 1. Pancreatic cyst fluid was safely collected via EUS-FNA (*n* = 10) and ERCP (*n* = 1) from all subjects. Five patients had asymptomatic BD-IPMN with the final diagnosis made by surgical pathology in three patients and radiology in two patients. These patients both had MRCP demonstrating communication of a nondilated main pancreatic duct with the cyst. The nondilated pancreatic duct was confirmed by EUS in both patients. One patient with mixed IPMN presented with acute pancreatitis. Final diagnosis of mixed IPMN was determined by pathology in four patients and radiology in one patient who refused surgery. The latter patient had a diffusely dilated main pancreatic duct to 7 mm with communication of the cystic lesion to this pancreatic duct seen on MRCP and EUS. As expected, amylase and CEA concentrations were not significantly different between BD-IPMN and mixed IPMN samples. 

### 3.2. Protein Microarray Assay Detected IMPs in All Ten Pancreatic Cyst Fluid Samples

**Figure 2:**
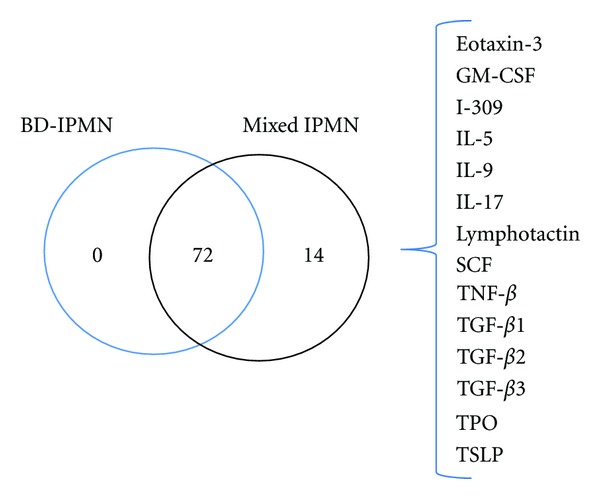
Venn diagram of IMPs identified in BD-IPMN and mixed IPMN. IMPs detected only in mixed IPMN are listed to the right of the diagram. Of the 89 IMPs assayed, three were not detected in either types of cyst (b-NGF, IL-11 and IL-29).

The concentration of IMPs ranged from below the limit of detection to greater than 15,000 pg/mL, and several IMPs had median concentrations above >1000 pg/mL. In the BD-IPMN samples, ENA-78 and NAP2 were detected with median concentrations greater than 1000 pg/mL. Similarly in the mixed IPMN samples, HCC1, ICAM1, MIF, NAP2, PDGF-AA, and SCGF-B had median concentrations greater than 1000 pg/mL.  Figure 2 summarizes the proteins detected in the BD-IPMN and mixed IPMN samples. Fourteen proteins were identified only in mixed IPMN fluid, while none of the assayed IMPs were solely found in BD-IPMN samples. In addition, 72 of the 89 IMPs assayed were present in both types of cysts (Supplementary Table 2), while 3 IMPs (b-NGF, IL-11 and IL-29) were not detected in either cohorts. 

### 3.3. Fourteen IMPs Were Detected Only in Mixed IPMN Fluid Aspirates (Table 2)

The following IMPs were all present in mixed IPMN and not detected in BD-IPMN samples: eotaxin 3, GM-CSF, I-309, IL-5, IL-9, IL-17, lymphotactin, TGF-*β*1, TGF-*β*2, TGF-*β*3, TNF-*β*, SCF, TPO, and TSLP. The concentrations of these IMPs in individual mixed IPMN samples ranged from 0.5 to 170.7 pg/mL. The majority of these IMPs was detected in one or two samples. TGF-*β*1, however, was identified in 3 samples. No IMPs were detected only in BD-IPMN cyst fluid.

### 3.4. Three IMPs Were Present in Higher Concentrations in Mixed IPMN Fluid Aspirates (Supplementary Table 2 and Figure 3)

Among the 72 IMPs detected in both BD-IPMN and mixed IPMN samples, G-CSF (*P* < 0.05), IL-23 (*P* < 0.1), and VCAM-1 (*P* < 0.1) had higher concentrations in mixed IPMN compared to BD-IPMN samples. None of the 72 proteins found in both BD-IPMN and mixed IPMN samples had significantly higher concentrations in BD-IPMN fluid samples.

## 4. Discussion

We identified IMPs with a microsphere-based suspension protein microarray assay in all endoscopically obtained pancreatic cyst fluid samples from patients with BD-IPMN and mixed IPMN. Our study differentiated the IMP profiles of BD-IPMN and mixed IPMN fluid aspirates. Specifically, we identified a total of 17 IMPs from the 89 tested that were differentially expressed between BD-IPMN and mixed IPMN. Fourteen IMPs were detected only in mixed IPMN, while three IMPs were present in higher concentrations in mixed IPMN. 

Accurate differentiation between BD-IPMN and mixed IPMN has important clinical implications. Mixed IPMN harbors a risk of malignancy up to 50–70%, similar to main duct IPMN (MD-IPMN), compared to approximately 15–25% for BD-IPMN; therefore, current guidelines recommend surgical resection of mixed IPMN [22]. In contrast, many BD-IPMN, including small ones without suspicious radiologic features, may be managed conservatively [22]. Differentiating MD-IPMN from BD-IPMN by radiologic criteria is clearly defined in the recent International Association of Pancreatology guidelines [23] while mixed IPMN may be more difficult to separate from BD-IPMN [24]. Therefore, we focused our study on differentiating BD-IPMN from mixed IPMN as additional tools are needed to accurately classify IPMNs. We believe the IMPs we have identified differentiating mixed IPMN from BD-IPMN merit further investigation as potential biomarkers of pancreatic cystic neoplasms. 

Cytokine and chemokine production is closely linked to pancreatic stellate cell function, particularly in the pathogenesis of pancreatic cancer [25–28]. Pancreatic stellate cells express growth factors, chemokines, and cytokines known to participate in inflammatory and fibrotic responses to pancreatic injury [25, 29–34]. These responses are often precursors to the development of malignant and pre-malignant lesions [35–38]. The expressed cytokines and chemokines controlling the cellular functions of pancreatic stellate cells represent potential diagnostic and therapeutic targets, several of which have been identified in our current analysis of pancreatic cyst fluid, collected primarily by EUS-FNA.

The TGF-*β* family, in particular TGF-*β*1, is the most promising potential biomarker for mixed IPMN, as it was detected in three of the five mixed IPMN samples and none of the BD-IPMN samples. TGF-*β* is a family of proteins that control proliferation, differentiation, and other functions in most cells [39]. It plays a role in immunity and cancer by arresting the cell cycle at the G1 stage to stop proliferation, induce differentiation, and/or promote apoptosis [40]. The TGF-*β* family consists of three members with similar peptide structures (TGF-*β*1, TGF-*β*2, and TGF-*β*3), all three of which were identified only in mixed IPMN samples. Interestingly, multiple studies have demonstrated an association between TGF-*β* and pancreatic cancer [41–48]. In addition, TGF-*β* signals through SMAD4, a critical tumor suppressor inactivated in half of pancreatic cancers [49].

G-CSF, IL-23, and VCAM-1 had higher expression levels in mixed IPMN compared to BD-IPMN and also represent potential diagnostic biomarkers. Additionally, these cytokines may lead to insights into the oncogenic nature of these pancreatic cystic neoplasms. Pancreatic cancer has been associated with elevated serum G-CSF [50, 51] and G-CSF positive immunohistochemistry [52]. G-CSF shares proinflammatory properties with IL-23, which we also measured in higher concentration in mixed IPMN [53]. IL-23 is produced by macrophages and thus has a role in the inflammatory response to infection and can promote tumor genesis and growth [54]. VCAM-1 mediates the adhesion of eosinophils, lymphocytes, monocytes, and basophils to vascular endothelium [55] and has been shown to be upregulated in pancreatic disease [56]; however, its role in mixed IPMN and cancer remains unclear. 

Our results demonstrate the applicability of IMP analysis in differentiating mixed IPMN from BD-IPMN, but must be validated further in larger studies. IMP profile comparisons with other clinically relevant pancreatic cystic lesions, including mucinous cystic neoplasms and serous cystadenomas, is needed and will expand the diagnostic utility of this technique. A potential limitation is that the peak concentration of certain IMPs in pancreatic cyst fluid may depend on the degree of dysplasia in the cyst. Assessing IMP levels in pancreatic cysts with different grades of dysplasia merits further study. In addition, one of our patients with mixed IPMN had a history of acute pancreatitis, which can have an effect on the IMP levels in the pancreatic fluid. The larger size of pancreatic cysts in the mixed IPMN may have affected IMP levels as well, and this needs further study.

In conclusion, we have successfully identified differentially expressed IMPs in pancreatic cyst fluid of BD-IPMN compared with mixed IPMN using endoscopic collection methods in tandem with cytokine microarray technology. With further validation, our findings may enable the accurate differentiation of mixed IPMN from BD-IPMN using a diagnostic cytokine panel. The advantages of directly investigating pancreatic cyst fluid with this microarray technology include high specificity, small sample volume requirement, cost effectiveness, and complementarity to other detection methods, such as mass spectrometry, ELISA, and western blotting [57]. Further investigation of other pancreatic cystic neoplasms, as well as the different degrees of dysplasia in various pancreatic cysts, using the methods described herein may generate major insights into cytokine-mediated pathogenesis of pancreatic cancer.

## Supplementary Material

Supplementary Table 1 lists the 89 IMPs with their corresponding abbreviations that were investigated in this study.
Supplementary Table 2 lists the 72 IMPs that were present in both BD-IPMN and mixed IPMN with their concentrations in each of the 10 fluid samples and the corresponding p-values.

## Figures and Tables

**Figure 3:**
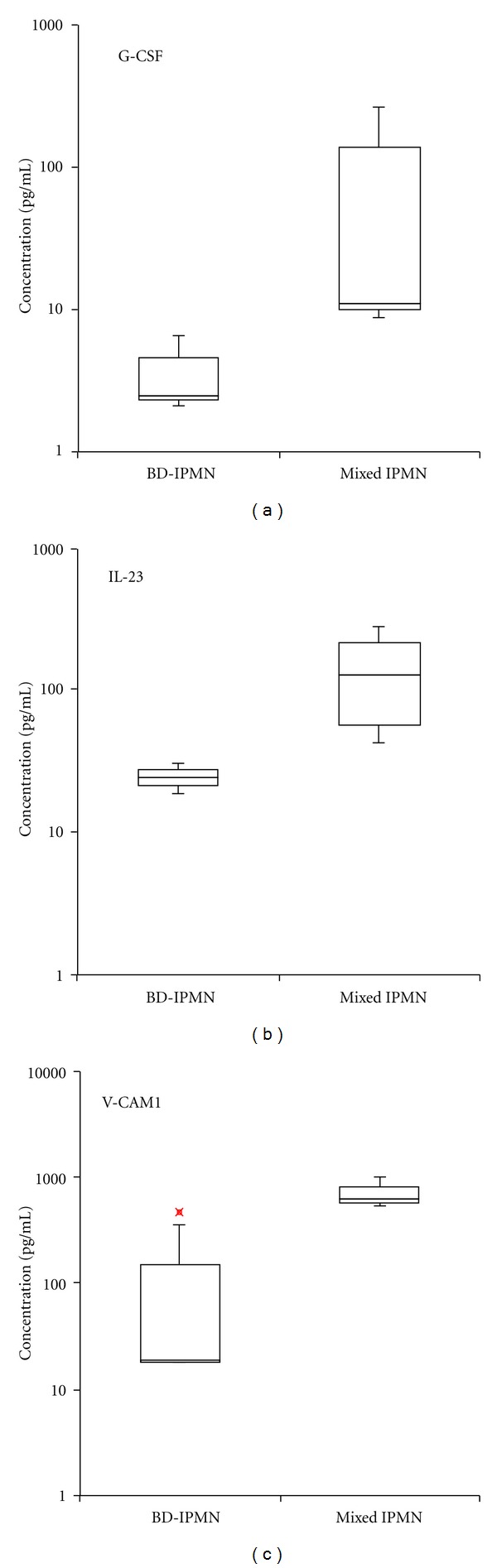
Box and whisker plots of differentially expressed IMPs between BD-IPMN and mixed IPMN. (a) G-CSF, *P* value < 0.05, (b) IL-23, and (c) VCAM-1, *P* values < 0.1. Bottom and top edges of box at 25th and 75th percentiles, respectively. Horizontal line within box marks 50th percentile (median). Whiskers extend from box as far as data extend, at most 1.5 interquartile ranges. Outlier represented by “x”.

**Table 1:** Patient characteristics.

Patient	Age (yr)	Gender	CEA (ng/mL)	Amylase (U/L)	Cytology	Imaging	Endoscopy	Surgical pathology
B1	81	Female	87.4	14700	EUS-FNA: no malignant cells	MRI: multiple septated cysts, largest 19 mm communicating with main PD 6 mm	EUS: multiple cysts, 17 mm communicating with main PD	BD-IPMN, moderate dysplasia
B2	61	Female	8.8	3	EUS-FNA: no malignant cells	MRI: 23 mm cyst communicating with nondilated main PD	EUS: 22 mm pancreatic cyst with nondilated 2 mm main PD	N/A
B3	60	Female	5980	40085	EUS-FNA: nondiagnostic	CT: 19 mm cyst communicating with nondilated main PD	EUS: 21 mm septated pancreatic cyst	BD-IPMN, LGD
B4	69	Male	2363	86.7	EUS-FNA: nondiagnostic	MRI: 19 mm septated cyst not communicating with nondilated main PD	EUS: 14 mm pancreatic cyst	BD-IPMN, LGD
B5	70	Female	2782	65	EUS-FNA: nondiagnostic	MRI: multiple septated cyst, largest 19 mm communicating with nondilated main PD	EUS: 13 mm pancreatic cyst with nondilated 1.6 mm main PD	N/A
M1	67	Female	632.2	37574	EUS-FNA of cysts in tail: atypical cells	MRI: multiple BD-IPMN with main PD dilation consistent with mixed IPMN	EUS: 3.2 × 1.8 cm cluster of cysts in tail	Mixed IPMN, HGD
M2	86	Female	N/A	N/A	ERCP: IPMN, LGD	MRI: diffusely dilated main PD, 5.2 cm cyst head/uncinate pancreas consistent with mixed IPMN	ERCP: mucus at papilla	Mixed IPMN, LGD
M3	85	Female	N/A	N/A	ERCP: adenocarcinoma	CT: diffuse MD-IPMN	EUS and ERCP: massively dilated main PD, mucus at papilla	Mixed IPMN, HGD, foci invasive adenocarcinoma
M4	78	Female	46	<10	EUS-FNA of cyst in body: no malignant cells	CT: 2.4 cm cyst in body of pancreas	EUS: 3.3 cm cyst in body	Mixed IPMN, LGD
M5	79	Male	20	N/A	EUS-FNA of cyst in uncinate: no malignant cells	CT and MRI: mixed IPMN with 4.8 cm cyst in head of pancreas and dilated main PD	EUS: 3.4 cm cyst in uncinate with dilated main PD 7 mm	N/A

N/A: not available; LGD: low grade dysplasia; HGD: high grade dysplasia.

**Table 2:** Inflammatory mediator proteins (*n* = 14) detected only in mixed IPMN.

Cytokine	Mixed IPMN						
	Concentration, pg/mL						
	Samples					Median	IQR
	M1	M2	M3	M4	M5		
Eotaxin-3	N.D.	91.2	11.8	N.D.	N.D.	51.5	39.7
GM-CSF	N.D.	127.5	9.3	N.D.	N.D.	68.4	59.1
I-309	N.D.	2.4	2.0	N.D.	N.D.	2.2	0.2
IL-5	N.D.	11.7	0.5	N.D.	N.D.	6.1	5.6
IL-9	N.D.	14.2	N.D.	N.D.	N.D.	14.2	0.0
IL-17	N.D.	15.7	6.7	N.D.	N.D.	11.2	4.5
Lymphotactin	N.D.	N.D.	N.D.	N.D.	37.2	37.2	0.0
SCF	N.D.	6.4	2.3	N.D.	N.D.	4.3	2.1
TGF-*β*1	N.D.	104.3	94.5	N.D.	81.2	94.5	11.5
TGF-*β*2	N.D.	170.7	41.4	N.D.	N.D.	106.0	64.7
TGF-*β*3	N.D.	14.7	N.D.	N.D.	N.D.	14.7	0.0
TNF-*β*	N.D.	3.8	8.6	N.D.	N.D.	6.2	2.4
TPO	N.D.	76.7	26.7	N.D.	N.D.	51.7	25.0
TSLP	N.D.	6.3	N.D.	N.D.	N.D.	6.3	0.0

IQR: interquartile range; N.D.: not detected.

## List of Abbreviations

BD-IPMN: Branch duct intraductal papillary mucinous neoplasm
MD-IPMN: Main duct intraductal papillary mucinous neoplasm
EUS-FNA: Endoscopic ultrasound-guided fine needle aspiration
ERCP: Endoscopic retrograde cholangiopancreatography
IMP: Inflammatory mediator protein
CT: Computed tomography
MRCP: Magnetic resonance cholangiopancreatography.

## References

[1] Lee LS, Saltzman JR, Bounds BC, Poneros JM, Brugge WR, Thompson CC (2005). EUS-guided fine needle aspiration of pancreatic cysts: a retrospective analysis of complications and their predictors. *Clinical Gastroenterology and Hepatology*.

[2] Al Haddad H, Laursen PB, Chollet D, Ahmaidi S, Buchheit M (2011). Reliability of resting and postexercise heart rate measures. *International Journal of Sports Medicine*.

[3] Maker AV, Lee LS, Raut CP, Clancy TE, Swanson RS (2008). Cytology from pancreatic cysts has marginal utility in surgical decision-making. *Annals of Surgical Oncology*.

[4] Cannon JG (2000). Inflammatory cytokines in nonpathological states. *News in Physiological Sciences*.

[5] Fernandez EJ, Lolis E (2002). Structure, function, and inhibition of chemokines. *Annual Review of Pharmacology and Toxicology*.

[6] Rottman JB (1999). Key role of chemokines and chemokine receptors in inflammation, immunity, neoplasia, and infectious disease. *Veterinary Pathology*.

[7] FitzGerald SP, McConnell RI, Huxley A (2008). Simultaneous analysis of circulating human cytokines using a high-sensitivity cytokine biochip array. *Journal of Proteome Research*.

[8] Paulo JA, Lee LS, Wu B, Banks PA, Steen H, Conwell DL (2011). Cytokine profiling of pancreatic fluid using the ePFT collection method in tandem with a multiplexed microarray assay. *Journal of Immunological Methods*.

[9] Sahani DV, Sainani NI, Blake MA, Crippa S, Mino-Kenudson M, Fernandez Del-Castillo C (2011). Prospective evaluation of reader performance on MDCT in characterization of cystic pancreatic lesions and prediction of cyst biologic aggressiveness. *American Journal of Roentgenology*.

[10] Kubo H, Chijiiwa Y, Akahoshi K (2001). Intraductal papillary-mucinous tumors of the pancreas: differential diagnosis between benign and malignant tumors by endoscopic ultrasonography. *American Journal of Gastroenterology*.

[11] Hruban RH, Takaori K, Klimstra DS (2004). An illustrated consensus on the classification of pancreatic intraepithelial neoplasia and intraductal papillary mucinous neoplasms. *American Journal of Surgical Pathology*.

[12] Reid-Lombardo KM, St Sauver J, Li Z, Ahrens WA, Unni KK, Que FG (2008). Incidence, prevalence, and management of intraductal papillary mucinous neoplasm in Olmsted County, Minnesota, 1984–2005: a Population Study. *Pancreas*.

[13] Paulo JA, Lee LS, Wu B (2010). Proteomic analysis of endoscopically (endoscopic pancreatic function test) collected gastroduodenal fluid using in-gel tryptic digestion followed by LC-MS/MS. *Proteomics-Clinical Applications*.

[14] Paulo JA, Lee LS, Wu B (2010). Optimized sample preparation of endoscopic collected pancreatic fluid for SDS-PAGE analysis. *Electrophoresis*.

[15] Paulo JA, Lee LS, Wu B (2010). Identification of pancreas-specific proteins in endoscopically (endoscopic pancreatic function test) collected pancreatic fluid with liquid chromatography-tandem mass spectrometry. *Pancreas*.

[16] Paulo JA, Kadiyala V, Lee LS, Banks PA, Conwell DL, Steen H (2012). Proteomic analysis (gelc-ms/ms) of epft-collected pancreatic fluid in chronic pancreatitis. *Journal of Proteome Research*.

[17] Opalka D, Lachman CE, MacMullen SA (2003). Simultaneous quantitation of antibodies to neutralizing epitopes on virus-like particles for human papillomavirus types 6, 11, 16, and 18 by a multiplexed luminex assay. *Clinical and Diagnostic Laboratory Immunology*.

[18] Carson RT, Vignali DAA (1999). Simultaneous quantitation of 15 cytokines using a multiplexed flow cytometric assay. *Journal of Immunological Methods*.

[19] Sachdeva N, Asthana D (2007). Cytokine quantitation: technologies and applications. *Frontiers in Bioscience*.

[20] Vignali DAA (2000). Multiplexed particle-based flow cytometric assays. *Journal of Immunological Methods*.

[21] Bender R, Lange S (2001). Adjusting for multiple testing—when and how?. *Journal of Clinical Epidemiology*.

[22] Tanaka M, Chari S, Adsay V (2006). International consensus guidelines for management of intraductal papillary mucinous neoplasms and mucinous cystic neoplasms of the pancreas. *Pancreatology*.

[23] Tanaka M, Fernandez-del Castillo C, Adsay V (2012). International consensus guidelines 2012 for the management of ipmn and mcn of the pancreas. *Pancreatology*.

[24] Pedrosa I, Boparai D (2010). Imaging considerations in intraductal papillary mucinous neoplasms of the pancreas. *World Journal of Gastrointestinal Surgery*.

[25] Shimizu K (2008). Mechanisms of pancreatic fibrosis and applications to the treatment of chronic pancreatitis. *Journal of Gastroenterology*.

[26] Patel M, Fine DR (2005). Fibrogenesis in the pancreas after acinar cell injury. *Scandinavian Journal of Surgery*.

[27] Ellenrieder V, Schneiderhan W, Bachem M, Adler G (2004). Fibrogenesis in the pancreas. *Roczniki Akademii Medycznej w Bialymstoku*.

[28] Apte MV, Wilson JS (2004). Mechanisms of pancreatic fibrosis. *Digestive Diseases*.

[29] Masamune A, Kikuta K, Watanabe T (2009). Fibrinogen induces cytokine and collagen production in pancreatic stellate cells. *Gut*.

[30] Farrow B, Albo D, Berger DH (2008). The role of the tumor microenvironment in the progression of pancreatic cancer. *Journal of Surgical Research*.

[31] Aust S, Jäger W, Kirschner H, Klimpfinger M, Thalhammer T (2008). Pancreatic stellate/myofibroblast cells express G-protein-coupled melatonin receptor 1. *Wiener Medizinische Wochenschrift*.

[32] Vonlaufen A, Apte MV, Imhof BA, Frossard JL (2007). The role of inflammatory and parenchymal cells in acute pancreatitis. *Journal of Pathology*.

[33] Mews P, Phillips P, Fahmy R (2002). Pancreatic stellate cells respond to inflammatory cytokines: potential role in chronic pancreatitis. *Gut*.

[34] Apte MV, Haber PS, Darby SJ (1999). Pancreatic stellate cells are activated by proinflammatory cytokines: implications for pancreatic fibrogenesis. *Gut*.

[35] Preis M, Korc : M (2011). Signaling pathways in pancreatic cancer. *Critical Reviews in Eukaryotic Gene Expression*.

[36] Masamune A, Watanabe T, Kikuta K, Shimosegawa T (2009). Roles of pancreatic stellate cells in pancreatic inflammation and fibrosis. *Clinical Gastroenterology and Hepatology*.

[37] Shimizu K (2008). Pancreatic stellate cells: molecular mechanism of pancreatic fibrosis. *Journal of Gastroenterology and Hepatology*.

[38] Jaster R, Emmrich J (2008). Crucial role of fibrogenesis in pancreatic diseases. *Best Practice and Research in Clinical Gastroenterology*.

[39] Blobe GC, Schiemann WP, Lodish HF (2000). Role of transforming growth factor *β* in human disease. *New England Journal of Medicine*.

[40] Khalil N (1999). TGF-*β*: from latent to active. *Microbes and Infection*.

[41] Guo-Yang WU, Qingjun LU, Hasenberg T (2010). Association between EGF, TGF-*β*l, TNF-*α* gene polymorphisms and cancer of the pancreatic head. *Anticancer Research*.

[42] Chow JYC, Ban M, Wu HL (2010). TGF-*β* downregulates PTEN via activation of NF-*κ*B in pancreatic cancer cells. *American Journal of Physiology-Gastrointestinal and Liver Physiology*.

[43] Truty MJ, Urrutia R (2007). Basics of TGF-*β* and pancreatic cancer. *Pancreatology*.

[44] Aoyagi Y, Oda T, Kinoshita T (2004). Overexpression of TGF-*β* by infiltrated granulocytes correlates with the expression of collagen mRNA in pancreatic cancer. *British Journal of Cancer*.

[45] Ellenrieder V, Buck A, Harth A (2004). KLF11 mediates a critical mechanism in TGF-*β* signaling that is inactivated by ERK-MAPK in pancreatic cancer cells. *Gastroenterology*.

[46] Teraoka H, Sawada T, Yamashita Y (2001). TGF-beta1 promotes liver metastasis of pancreatic cancer by modulating the capacity of cellular invasion. *International Journal of Oncology*.

[47] Ellenrieder V, Hendler SF, Ruhland C, Boeck W, Adler G, Gress TM (2001). TGF-*β*-induced invasiveness of pancreatic cancer cells is mediated by matrix metalloproteinase-2 and the urokinase plasminogen activator system. *International Journal of Cancer*.

[48] Kleeff J, Ishiwata T, Maruyama H (1999). The TGF-*β* signaling inhibitor Smad7 enhances tumorigenicity in pancreatic cancer. *Oncogene*.

[49] Zavoral M, Minarikova P, Zavada F, Salek C, Minarik M (2011). Molecular biology of pancreatic cancer. *World Journal of Gastroenterology*.

[50] Groblewska M, Mroczko B, Wereszczynska-Siemiatkowska U, Mysliwiec P, Kedra B, Szmitkowski M (2007). Serum levels of granulocyte colony-stimulating factor (G-CSF) and macrophage colony-stimulating factor (M-CSF) in pancreatic cancer patients. *Clinical Chemistry and Laboratory Medicine*.

[51] Joshita S, Nakazawa K, Sugiyama Y (2009). Granulocyte-colony stimulating factor-producing pancreatic adenosquamous carcinoma showing aggressive clinical course. *Internal Medicine*.

[52] Ohtsubo K, Mouri H, Sakai J (1998). Pancreatic cancer associated with granulocyte-colony stimulating factor production confirmed by immunohistochemistry. *Journal of Clinical Gastroenterology*.

[53] Wiekowski MT, Leach MW, Evans EW (2001). Ubiquitous transgenic expression of the IL-23 subunit p19 induces multiorgan inflammation, runting, infertility, and premature death. *Journal of Immunology*.

[54] Langowski JL, Zhang X, Wu L (2006). IL-23 promotes tumour incidence and growth. *Nature*.

[55] Barreiro O, Yáñez-Mó M, Serrador JM (2002). Dynamic interaction of VCAM-1 and ICAM-1 with moesin and ezrin in a novel endothelial docking structure for adherent leukocytes. *Journal of Cell Biology*.

[56] Kleinhans H, Kaifi JT, Mann O (2009). The role of vascular adhesion molecules PECAM-1 (CD 31), VCAM-1 (CD 106), E-selectin (CD62E) and P-selectin (CD62P) in severe porcine pancreatitis. *Histology and Histopathology*.

[57] Pollard HB, Srivastava M, Eidelman O (2007). Protein microarray platforms for clinical proteomics. *Proteomics-Clinical Applications*.

