# Supplementary material for: Differentiating Branch Duct and Mixed IPMN in Endoscopically Collected Pancreatic Cyst Fluid via Cytokine Analysis
Source: Gastroenterol Res Pract. 2012 Dec 25;2012:247309. doi: 10.1155/2012/247309 (PMC3543798; doi:10.1155/2012/247309)
Supplement: Supplementary file 1 — Supplementary Table 1 lists the 89 IMPs with their corresponding abbreviations that were investigated in this study. Supplementary Table 2 lists the 72 IMPs that were present in both BD-IPMN and mixed IPMN with their concentrations in each of the 10 fluid samples and the corresponding p-values. [file 247309.f1.pdf]

Supplemental Table 1: Panel of inflammatory mediator proteins investigated in this study.

Abbrev.	Full Protein Name	UniProt #
EGF	Epidermal Growth Factor	P01133
6Ckine	Chemokine with 6 cysteines	O00585
BCA-1	B cell-attracting chemokine 1	O43927
b-NGF	Beta-nerve growth factor	P01138
CTACK	Cutaneous T-cell-attracting chemokine	Q9Y4X3
CXCL6	C-X-C motif chemokine ligand 6	P80162
ENA-78	Epithelial-derived neutrophil-activating protein 78	P42830
Eotaxin	Eosinophil chemotactic protein	P51671
Eotaxin-2	Eosinophil chemotactic protein 2	O00175
Eotaxin-3	Eosinophil chemotactic protein 2	Q9Y258
FGF-basic	Basic fibroblast growth factor 2	P09038
Flt-3L	Fms-related tyrosine kinase 3 ligand	P49771
Fractalkine	Fractalkine	P78423
G-CSF	Granulocyte colony-stimulating factor	P09919
GM-CSF	Granulocyte-macrophage colony-stimulating factor	P04141
GRO	Growth-regulated protein beta	P19875
GRO-a	Growth-regulated protein alpha	P09341
HCC-1	C-C motif chemokine 14	Q16627
HGF	Hepatocyte growth factor	P14210
I-309	T lymphocyte-secreted protein I-309	P22362
ICAM-1	Intercellular adhesion molecule 1	P05362
IFN-a2	Interferon alpha-2protein	Q6DJX8
IFN-g	Interferon gamma	P01579
IL-10	Interleukin-10	P22301
IL-11	Interleukin-11	P20809
IL-12p40	Interleukin-12 subunit	Q8N0X8
IL-12p70	Interleukin-12 subunit	Q8N0X8
IL-13	Interleukin-13	P35225
IL-15	Interleukin-15	P40933
IL-16	Interleukin-16	Q14005
IL-17	Interleukin-17	Q5QEX9
IL-18	Interleukin-18	Q14116
IL-1a	Interleukin-1 alpha	Q53QF9
IL-1b	Interleukin-1 beta	P01584
IL-1ra	Interleukin-1 receptor antagonist protein	P18510
IL-2	Interleukin-2	P60568
IL-20	Interleukin-20	Q9NYY1
IL-21	Interleukin-21	Q9HBE4
IL-23	Interleukin-23	Q9NPF7
IL-28A	Interleukin-28 subunit alpha	Q8IZJ0
IL-29	Interleukin-29	Q8IU54
IL-3	Interleukin-3	P08700
IL-33	Interleukin-33	O95760
IL-4	Interleukin-4	P05112
IL-5	Interleukin-5	P05113
IL-6	Interleukin-6	P05231
IL-7	Interleukin-7	P13232
IL-8	Interleukin-8	P10145
IL-9	Interleukin-9	P15248
IP-10	10 kDa interferon gamma-induced protein	P02778
I-TAC	Interferon-inducible T-cell alpha chemoattractant	O14625
LIF	Leukemia inhibitory factor	P15018
Lymphotactin	Lymphotactin	P47992
MCP-1	Monocyte chemotactic protein 1	P13500
MCP-2	Monocyte chemotactic protein 2	P80075
MCP-3	Monocyte chemotactic protein 3	P80098
MCP-4	Monocyte chemotactic protein 4	Q99616
M-CSF	Macrophage colony-stimulating factor 1	P09603
MDC	Macrophage-derived chemokine	O00626
MIF	Macrophage migration inhibitory factor	P14174
MIG	Monokine induced by interferon-gamma	Q07325
MIP-1a	Macrophage inflammatory protein 1-alpha	P10147
MIP-1b	Macrophage inflammatory protein 1-beta	P13236
MIP-1d	Macrophage inflammatory protein delta	Q16663
MIP-3a	Macrophage inflammatory protein 3-alpha	P78556
MIP-3b	Macrophage inflammatory protein 3-beta	Q99731
NAP-2	Nucleosome assembly protein 2	Q99733
PDGF-AA	Platelet-derived growth factor subunit A	P04085
PDGF-AB/BB	Platelet-derived growth factor subunit A and B	P04085
PDGF-BB	Platelet-derived growth factor subunit B	P01127
Rantes	Rantes	P13501
sCD40L	Cluster of Differentiation-40 ligand	P29965
SCF	Stem cell factor	P21583
SCGF-b	Stem cell growth factor-beta	Q9Y240
SDF-1a	Stromal cell-derived factor 1 isoform A	P48061
SDF-1a+b	Stromal cell-derived factor 1 isoform A and B	P48061
sIL-2Ra	Soluble Interleukin-2 receptor subunit alpha	P01589
TARC	Thymus and activation-regulated chemokine	Q92583
TGF-a	Transforming growth factor alpha	P01135
TGF-b1	Transforming growth factor beta-1	P01137
TGF-b2	Transforming growth factor beta-2	P61812
TGF-b3	Transforming growth factor beta-3	P10600
TNF-a	Tumor necrosis factor-alpha	P01375
TNF-b	Tumor necrosis factor-beta	P01374
TPO	Thyroid peroxidase	P07202
TRAIL	TNF-related apoptosis-inducing ligand	P50591
TSLP	Thymic stromal lymphopoietin	Q969D9
VCAM-1	Vascular cell adhesion protein 1	P19320
VEGF	Vascular endothelial growth factor A	P15692
