# Supplementary material for: Differentiating Branch Duct and Mixed IPMN in Endoscopically Collected Pancreatic Cyst Fluid via Cytokine Analysis
Source: Gastroenterol Res Pract. 2012 Dec 25;2012:247309. doi: 10.1155/2012/247309 (PMC3543798; doi:10.1155/2012/247309)
Supplement: Supplementary file 2 [file 247309.f2.pdf]

Supplemental Table 2: Statistical analysis of IMPs detected in both BD-IPMN and mixed IPMN samples.

Cytokine	BD-IPMN							Mixed IPMN							p-value	
	Concentration, pg/ml							Concentration, pg/ml								
	Samples					Median	IQR	Samples					Median	IQR		
	B1	B2	B3	B4	B5			M1	M2	M3	M4	M5				
6Ckine	N.D.	N.D.	104.4	N.D.	N.D.	104.4	0.0	N.D.	208.3	64.1	N.D.	40.5	64.1	83.9	0.6547	
BCA-1	N.D.	N.D.	0.7	N.D.	N.D.	0.7	0.0	N.D.	20.0	1.8	N.D.	N.D.	10.9	9.1	0.2207	
CTACK	N.D.	1.0	2.7	N.D.	N.D.	1.9	0.9	N.D.	0.9	N.D.	N.D.	N.D.	0.9	0.0	0.2207	
CXCL6	N.D.	N.D.	32.3	7.4	N.D.	19.8	12.4	N.D.	2.1	4.3	16.2	5.9	5.1	4.7	0.1649	
EGF	5.9	363.7	1305.5	N.D.	N.D.	363.7	649.8	11620.2	47.4	19.0	108.9	N.D.	78.1	2946.4	1.0000	
ENA-78	N.D.	N.D.	2566.7	139.5	N.D.	1353.1	1213.6	N.D.	1082.8	43.1	369.7	279.4	324.6	327.6	0.6434	
Eotaxin	2.8	18.6	18.2	8.6	N.D.	13.4	11.1	12.3	38.6	24.0	N.D.	N.D.	24.0	13.1	0.1573	
Eotaxin-2	13.7	36.0	1741.8	8.0	117.9	36.0	104.2	147.5	2521.2	6666.7	12.8	350.3	350.3	2373.7	0.1745	
FGF-basic	N.D.	54.5	39.8	N.D.	N.D.	47.1	7.3	N.D.	201.5	45.7	N.D.	N.D.	123.6	77.9	0.4386	
Flt-3L	N.D.	3.4	N.D.	N.D.	N.D.	3.4	0.0	N.D.	79.5	18.1	N.D.	N.D.	48.8	30.7	0.2207	
Fractalkine	26.0	46.2	168.2	26.0	214.0	46.2	142.2	26.0	3120.0	533.1	N.D.	158.4	345.7	1054.5	0.2967	
G-CSF	N.D.	2.1	6.6	2.5	N.D.	2.5	2.2	N.D.	266.4	11.2	N.D.	8.8	11.2	128.8	0.0495	*
GRO	N.D.	50.2	658.1	15.9	15.9	33.0	186.2	11.5	1174.4	383.1	603.4	243.4	383.1	360.0	0.4624	
GRO-a	N.D.	24.6	312.4	7.8	7.8	16.2	88.8	6.2	607.3	197.3	299.6	119.2	197.3	180.4	0.4624	
HCC-1	N.D.	171.8	818.7	N.D.	39.2	171.8	389.7	N.D.	1563.4	1705.9	15.7	1316.4	1439.9	607.8	0.2888	
HGF	27.8	144.1	474.3	20.3	12.6	27.8	123.8	329.1	4797.0	448.2	76.0	40.6	329.1	372.2	0.1745	
ICAM-1	276.6	4492.5	5375.7	276.6	259.0	276.6	4215.9	18274.0	16409.0	4353.5	3938.1	654.3	4353.5	12470.9	0.1745	
IFN-a2	N.D.	N.D.	12.9	N.D.	N.D.	12.9	0.0	N.D.	312.2	51.5	N.D.	N.D.	181.8	130.4	0.2207	
IFN-g	N.D.	2.3	1.1	1.4	N.D.	1.4	0.6	N.D.	236.0	14.3	N.D.	0.4	14.3	117.8	0.5127	
IL-10	0.2	1.9	2.9	N.D.	N.D.	1.9	1.4	N.D.	80.5	10.0	0.6	N.D.	10.0	40.0	0.2752	
IL-12p40	N.D.	17.5	7.0	N.D.	N.D.	12.2	5.2	N.D.	946.1	63.3	N.D.	N.D.	504.7	441.4	0.1213	
IL-12p70	N.D.	N.D.	2.0	N.D.	N.D.	2.0	0.0	N.D.	49.4	7.7	N.D.	N.D.	28.6	20.8	0.2207	
IL-13	N.D.	12.2	5.5	N.D.	N.D.	8.8	3.3	N.D.	211.5	11.5	N.D.	2.2	11.5	104.6	1.0000	
IL-15	N.D.	7.4	N.D.	N.D.	N.D.	7.4	0.0	N.D.	250.3	12.0	N.D.	N.D.	131.2	119.1	0.2207	
IL-16	7.3	11.8	28.1	20.2	2.4	11.8	12.9	16.1	270.1	18.2	11.8	N.D.	17.1	66.1	0.3913	
IL-18	N.D.	18.4	92.0	N.D.	N.D.	55.2	36.8	N.D.	92.7	8.0	N.D.	N.D.	50.3	42.4	1.0000	
IL-1a	N.D.	7.4	12.5	N.D.	N.D.	9.9	2.6	4.1	26.6	22.4	N.D.	N.D.	22.4	11.2	0.5637	
IL-1b	N.D.	1.0	N.D.	N.D.	N.D.	1.0	0.0	N.D.	1140.2	8.6	N.D.	N.D.	574.4	565.8	0.2207	
IL-1ra	N.D.	13.3	379.7	N.D.	N.D.	196.5	183.2	N.D.	595.6	46.2	N.D.	N.D.	320.9	274.7	0.4386	
IL-2	N.D.	1.2	2.1	N.D.	N.D.	1.6	0.5	N.D.	288.2	14.3	N.D.	N.D.	151.2	137.0	0.1213	

**IL-20**	N.D.	N.D.	50.2	N.D.	N.D.	50.2	0.0	N.D.	442.7	232.1	N.D.	N.D.	337.4	105.3	0.2207	
**IL-21**	N.D.	4.1	N.D.	N.D.	N.D.	4.1	0.0	N.D.	4.4	N.D.	N.D.	N.D.	4.4	0.0	0.3173	
**IL-23**	N.D.	18.3	30.2	N.D.	N.D.	24.2	5.9	59.7	273.8	42.0	188.8	N.D.	124.3	154.8	0.0641	**
**IL-28A**	2.9	N.D.	N.D.	N.D.	N.D.	2.9	0.0	N.D.	25.7	4.4	N.D.	N.D.	15.1	10.6	0.2207	
**IL-3**	N.D.	8.5	N.D.	2.6	N.D.	5.5	2.9	18.7	4.1	N.D.	N.D.	N.D.	11.4	7.3	0.4386	
**IL-33**	N.D.	N.D.	16.3	N.D.	N.D.	16.3	0.0	N.D.	19.3	16.3	N.D.	N.D.	17.8	1.5	0.5403	
**IL-4**	N.D.	2.2	0.7	16.5	N.D.	2.2	7.9	N.D.	351.2	9.4	N.D.	N.D.	180.3	170.9	0.2482	
**IL-6**	N.D.	5.4	21.9	N.D.	N.D.	13.6	8.2	N.D.	211.5	33.7	N.D.	N.D.	122.6	88.9	0.1213	
**IL-7**	N.D.	3.5	27.0	N.D.	2.7	3.5	12.1	N.D.	138.0	23.6	5.1	9.1	16.3	44.1	0.2888	
**IL-8**	9.3	7.7	617.7	N.D.	N.D.	9.3	305.0	23.3	9160.5	3067.2	14.0	5.7	23.3	3053.3	0.4561	
**IP-10**	6.1	24.2	106.0	4.4	N.D.	15.1	38.9	7.6	1457.4	419.8	11.3	N.D.	215.6	668.8	0.2482	
**I-TAC**	N.D.	0.9	373.7	N.D.	N.D.	187.3	186.4	N.D.	9.6	11.9	16.0	1.3	10.8	5.4	1.0000	
**LIF**	N.D.	15.0	N.D.	N.D.	N.D.	15.0	0.0	N.D.	224.2	N.D.	N.D.	N.D.	224.2	0.0	0.3173	
**MCP-1**	65.0	2591.2	631.6	2.3	1.0	65.0	629.3	4800.4	322.9	5965.9	153.2	10.8	322.9	4647.2	0.2506	
**MCP-2**	N.D.	N.D.	5.8	N.D.	N.D.	5.8	0.0	N.D.	20.5	25.9	N.D.	N.D.	23.2	2.7	0.2207	
**MCP-3**	4.3	5.7	11.4	2.0	2.0	4.3	3.7	N.D.	133.3	76.2	2.0	4.3	40.3	86.7	0.3913	
**MCP-4**	N.D.	N.D.	31.8	N.D.	N.D.	31.8	0.0	N.D.	126.5	162.9	N.D.	N.D.	144.7	18.2	0.2207	
**M-CSF**	15.4	N.D.	N.D.	14.2	N.D.	14.8	0.6	12.0	53.8	N.D.	N.D.	N.D.	32.9	20.9	1.0000	
**MDC**	N.D.	6.1	29.5	N.D.	N.D.	17.8	11.7	N.D.	585.8	82.6	N.D.	N.D.	334.2	251.6	0.1213	
**MIF**	162.8	598.6	12262.0	65.5	293.7	293.7	435.8	91.2	24973.9	11500.4	78.5	3863.1	3863.1	11409.3	0.6015	
**MIG**	N.D.	42.0	194.8	N.D.	16.2	42.0	89.3	143.8	2021.2	3912.9	54.1	82.4	143.8	1938.8	0.1797	
**MIP-1a**	N.D.	7.7	13.2	N.D.	N.D.	10.4	2.7	N.D.	320.6	24.1	N.D.	N.D.	172.3	148.2	0.1213	
**MIP-1b**	N.D.	32.9	31.4	N.D.	N.D.	32.1	0.7	20.2	308.4	53.5	N.D.	N.D.	53.5	144.1	0.5637	
**MIP-1d**	N.D.	N.D.	N.D.	N.D.	96.2	96.2	0.0	N.D.	2909.5	22.1	N.D.	63.4	63.4	1443.7	0.6547	
**MIP-3a**	N.D.	N.D.	47.3	N.D.	N.D.	47.3	0.0	N.D.	72.1	12.7	3.5	4.3	8.5	23.4	0.4795	
**MIP-3b**	N.D.	N.D.	2.1	N.D.	N.D.	2.1	0.0	N.D.	2.7	N.D.	N.D.	N.D.	2.7	0.0	0.3173	
**NAP-2**	N.D.	2236.6	5415.8	N.D.	N.D.	3826.2	1589.6	N.D.	N.D.	2217.3	N.D.	6116.7	4167.0	1949.7	1.0000	
**PDGF-AA**	152.6	9544.1	9784.4	35.4	130.3	152.6	9413.8	10457.0	7842.8	8056.4	644.5	276.9	7842.8	7411.8	0.3472	
**PDGF-AB/BB**	16.2	279.7	589.2	31.2	9.7	31.2	263.4	70.5	371.0	64.9	15.7	116.2	70.5	51.3	0.7540	
**PDGF-BB**	15.3	90.4	396.9	29.2	8.5	29.2	75.1	51.0	50.1	36.9	12.6	66.4	50.1	14.0	0.9168	
**Rantes**	3.4	218.4	1621.1	N.D.	N.D.	218.4	808.8	1.6	81.0	53.2	N.D.	980.0	67.1	265.5	0.4795	
**sCD40L**	N.D.	86.2	243.4	1.9	N.D.	86.2	120.8	1.9	23.9	111.4	1.9	55.9	23.9	54.0	0.4561	
**SCGF-b**	69.5	990.9	481.7	613.5	N.D.	547.6	329.2	1529.6	3290.4	1456.6	14.3	N.D.	1493.1	873.7	0.2482	
**SDF-1a**	54.3	59.1	338.1	76.2	38.5	59.1	21.9	49.2	260.6	49.2	38.5	4.8	49.2	10.7	0.2101	
**SDF-1a+b**	N.D.	452.5	427.3	78.1	N.D.	427.3	187.2	70.1	407.7	175.7	N.D.	N.D.	175.7	168.8	0.2752	
**sIL-2Ra**	N.D.	7.6	N.D.	N.D.	N.D.	7.6	0.0	N.D.	212.3	17.7	N.D.	N.D.	115.0	97.3	0.2207	

**TARC**	N.D.	0.4	2.8	N.D.	N.D.	1.6	1.2	0.4	5.3	2.0	N.D.	0.8	1.4	2.1	0.8170	
**TGF-a**	N.D.	N.D.	7.3	N.D.	1.3	4.3	3.0	N.D.	32.7	4.8	N.D.	1.9	4.8	15.4	0.5637	
**TNF-a**	N.D.	N.D.	1.4	N.D.	N.D.	1.4	0.0	N.D.	0.6	2.8	N.D.	N.D.	1.7	1.1	1.0000	
**TRAIL**	N.D.	2.0	49.3	N.D.	51.6	49.3	24.8	1.0	64.8	20.5	20.4	13.7	20.4	6.9	0.6547	
**VCAM-1**	N.D.	18.7	555.2	18.1	18.2	18.5	134.6	N.D.	618.5	1026.9	N.D.	539.2	618.5	243.9	0.0771	**
**VEGF**	N.D.	37.3	650.4	N.D.	14.0	37.3	318.2	N.D.	881.2	622.9	19.1	386.2	504.6	393.1	0.4795	

IQR, interquartile range; * = p-value<0.05; ** = p-value<0.1
